# Winners of the 2024 Tu Youyou Award

**DOI:** 10.3390/molecules30102192

**Published:** 2025-05-16

**Authors:** RuAngelie Edrada-Ebel, Angelo Fontana, Hideaki Kakeya, A. Douglas Kinghorn, Wei Li, Diana C. G. A. Pinto, Thomas E. Prisinzano, Binghe Wang

**Affiliations:** 1 Natural Products Metabolomics Group, Strathclyde Institute of Pharmacy and Biomedical Sciences, University of Strathclyde, Glasgow G4 0RE, UK; ruangelie.edrada-ebel@strath.ac.uk; 2 Department of Biology, University of Naples “Federico II”, 80126 Naples, Italy; angelo.fontana@unina.it; 3 Institute of Biomolecular Chemistry CNR, 80078 Naples, Italy; 4 Department of System Chemotherapy and Molecular Sciences, Division of Medicinal Frontier Sciences, Graduate School of Pharmaceutical Sciences, Kyoto University, Kyoto 606-8501, Japan; scseigyo-hisyo@pharm.kyoto-u.ac.jp; 5 Division of Medicinal Chemistry and Pharmacognosy, College of Pharmacy, The Ohio State University, Columbus, OH 43210, USA; 6 Department of Pharmaceutical Sciences, University of Tennessee Health Science Center, Memphis, TN 38163, USA; wli@uthsc.edu; 7 LAQV/REQUIMTE, Department of Chemistry, Campus Universitário de Santiago, University of Aveiro, 3810-193 Aveiro, Portugal; diana@ua.pt; 8 Department of Pharmaceutical Sciences, University of Kentucky, Lexington, KY 40506, USA; prisinzano@uky.edu; 9 Department of Chemistry and Center for Diagnostics and Therapeutics, Georgia State University, Atlanta, GA 30301, USA; wang@gsu.edu

Professor Tu Youyou is a renowned Chinese scientist whose pioneering work led to the discovery in the 1970s of the antimalarial sesquiterpene lactone, artemisinin (*qinghaosu*), from the sweet wormwood tree, *Artemisia annua* L. (Asteraceae). Artemisinin-based combination therapies are now widely used as frontline therapy in the treatment of malaria, as recommended by the World Health Organization [1]. For her seminal discovery of artemisinin, in 2015, Prof. Tu was selected as a co-recipient of the Nobel Prize in Physiology or Medicine. She has received several further major awards and was very recently elected as an International Member of the U.S. National Academy of Sciences [2].

The Tu Youyou Award was created by MDPI to honor outstanding work in natural product chemistry and medicinal chemistry. The previous winners include Kenneth A. Jacobsen (National Institutes of Health, Bethesda, MD, USA, 2016), Barry Potter (University of Oxford, United Kingdom, 2018), Mauro Maccarrone (University of L’Aquilla, Italy, 2020), and Xiaoguang Lei (Peking University, China, 2022). For the 2024 Yu Youyou Award, the total prize amount was increased to 100,000 CHF, to be shared equally among the awardees if more than one winner were selected. Nominations from an institution or scientific society were invited from April 2024 to 31 December 2024. Then, an international Awards Committee team evaluated each nomination. A two-stage evaluation process was undertaken; the nominees with the highest initial rankings were then discussed in detail in a subsequent round-table virtual meeting. Committee members with a conflict of interest did not participate in any ranking or voting for a given nominee. Following this process, Professors Rolf Müller (Saarland University, Saarbrücken, Germany) and Richard D. DiMarchi (Indiana University, Bloomington, IN, USA) were selected as the winners of the 2024 Tu Youyou Award.

Professor Rolf Müller is the Managing Director of the Helmholtz Institute for Pharmaceutical Research Saarland (HIPS), in addition to being Professor of Pharmaceutical Biotechnology and Head of the Department of Microbial Natural Products at Saarland University, Saarbrüken, Germany. After initially studying Pharmacy, he earned a Ph.D. in Pharmaceutical Biology from the University of Bonn in 1994. He then performed postdoctoral work both in Bonn and at the University of Washington, Seattle, WA, USA, prior to becoming a Junior Group Leader in Braunchsweig, Germany (1998–2003). Prof. Müller completed his Habilitation at the Technical University of Braunschweig in 2000 and was appointed as Full Professor at his present institution in 2003. He runs a prolific research program dealing with microbial natural product isolation and structural characterization, biosynthesis, genomics, industrial microbiology, and biotechnology. He has focused his drug discovery efforts mainly on new antibiotic development, including corollapyronin A and the darobactins. Professor Müller has been the recipient of several major honors, including the Gottfried Wilhelm Leibniz Prize of the German Research Foundation (2021) and the Charles Thom Award of the Society for Industrial Microbiology and Biotechnology, Fairfax, VA, USA (2023), and is an elected member of the German Academy of Sciences Leopoldina. A short selection of the published work of Professor Müller and his collaborators over the period 2009–2024 is cited below [3,4,5,6,7,8,9,10].

Professor Richard D. DiMarchi trained at the undergraduate level in Chemistry before receiving a Ph.D. in Biochemistry from Indiana University in 1979. He then carried out postdoctoral work at Rockefeller University in New York (1979–1981). Subsequently, he held positions of increasing seniority at the Lilly Research Laboratories, Indianapolis, IN, culminating in the role of Group Vice President (1996–2003). In 2003, he was appointed to his present position as Distinguished Professor of Chemistry and Gill Chair in Biomolecular Science at Indiana University, and, in addition, he took on the role of Vice President and Head of the Novo Nordisk Research Center, Indianapolis, during the period 2016–2020. Professor DiMarchi’s research program examines the medicinal chemistry of naturally occurring peptides and proteins in relation to endocrine diseases. Specific areas in which he has worked include investigating structure–function relationships in insulin, which led to the development of Humulin^®^ as the first rDNA-based medicine, and later on to Humalog^®^ (LisPro-human insulin). He was also instrumental, as a key investigator, in rGlucagon^®^ and Forteo^®^ being approved as drugs. A further major discovery by Professor DiMarchi, along with the late Professor Suad Efendic, was that GLP-1 treatment could decrease body weight in obese patients with type 2 diabetes. He has been accorded several major honors, including being inducted in 2015 into both the U.S. National Academy of Medicine and the U.S. National Academy of Inventors and, in 2023, the receipt of the American Association for the Advancement of Science Mani L. Bhaumik Breakthrough of the Year Award. Selected publications by Professor DiMarchi and his associates between 1997 and 2024 are provided in the bibliography of this Editorial [11,12,13,14,15,16,17,18].

In summary, it has been a great pleasure and an honor for us, the authors, to serve on the scientific evaluation committee for the 2024 Youyou Award. It is our collective opinion that the exceptional research contributions of Professors Müller and DiMarchi in natural product chemistry and medicinal chemistry, respectively, truly justify their selection as the recipients of this very prestigious honor.
